# *Trps1* Differentially Modulates the Bone Mineral Density between Male and Female Mice and Its Polymorphism Associates with BMD Differently between Women and Men

**DOI:** 10.1371/journal.pone.0084485

**Published:** 2014-01-08

**Authors:** Lishi Wang, Wenli Lu, Lei Zhang, Yue Huang, Rachel Scheib, Xiaoyun Liu, Linda Myers, Lu Lu, Charles R. Farber, Gaifen Liu, Cong-Yi Wang, Hongwen Deng, Robert W. Williams, Yongjun Wang, Weikuan Gu, Yan Jiao

**Affiliations:** 1 Department of Orthopedic Surgery and BioMedical Engineering, Campbell-Clinic, University of Tennessee Health Science Center, Memphis, Tennessee, United States of America; 2 Department of Basic Medicine, Inner Mongolia Medical University, Huhhot, Inner Mongolia, P.R. China; 3 Department of Biostatistics and Bioinformatics, School of Public Health and Tropical Medicine, Tulane University, New Orleans, Louisiana, United States of America; 4 Department of Medicine, University of Tennessee Health Science Center, Memphis, Tennessee, United States of America; 5 Department of Anatomy and Neurobiology, University of Tennessee Health Science Center, Memphis, Tennessee, United States of America; 6 Center for Public Health Genomics, Departments of Medicine (Division of Cardiology) and Biochemistry and Molecular Genetics, University of Virginia, Charlottesville, Virginia, United States of America; 7 Department of Neurology, Beijing Tiantan Hospital, Capital Medical University, Beijing, China; 8 The Center for Biomedical Research, Tongji Hospital, Tongji Medical College, Huazhong University of Science and Technology, Wuhan, HuBei, China; University of Chicago, United States of America

## Abstract

The objective of our study was to identify genetic factors that regulate bone mineral density (BMD) in mice using well defined recombinant inbred strains. For this purpose we chose the BXD recombinant inbred (RI) strains derived from progeny of the C57BL/6J (B6) and DBA/2J (D2) progenitor strains. We sampled both male and female mice (∼4 each) of 46 strains at 3 months-of-age, measured their BMD, and conducted QTL mapping. The data were analyzed to identify candidates genes contained within the most significant quantitative trait locus (QTL). Evaluation of candidate genes included functional assessment, single nucleotide polymorphism (SNP) genotyping and direct sequencing. We established that there was a QTL for BMD in males on chromosome 15 that has the impact larger than QTLs on all other chromosomes. The QTL on chromosome 15 was narrowed to a genomic region between 38 Mbp and 52 Mbp. By examining transcripts within this region, we found an important candidate gene: trichorhinophalangeal syndrome, type I (*Trps1*). SNP analysis identified a nonsynonymous SNP (*rs32398060*) in *Trps1* that co-segregated with bone mineral density. Analysis of association between this SNP within TRPS1 and BMD in a human population confirmed its significance.

## Introduction

Osteoporosis is a disease characterized by low bone mass primarily due to microarchitectural changes in trabecular bone. Bone mineral density (BMD) is the major genetic determinant for osteoporosis [2]–[5]. Low BMD is characterized by enhanced bone fragility and a consequent increase in fracture risk. In spite of the contribution of environmental factors, such as diet, drugs, exercise and coexisting diseases, peak BMD is predominantly determined by genetic influences. In fact genetic background has been estimated to account for more than 70% of variation in BMD. Thus, the identification of the gene/genes that regulate BMD may represent a major advance in understanding the pathogenesis of osteoporosis.

Many quantitative trait loci (QTL) influencing mouse model phenotypes for BMD have been genetically mapped [1]. However, the gene(s) comprising the QTL (QTGs) are largely unknown. Identification of QTGs has been a bottleneck in QTL studies not only for BMD but also for other complex traits. Resolution of this problem would allow characterization and understanding of the functional pathways underlying complex traits and ultimately implementation of treatments for complex diseases. Thus the approach described here may permit elucidation of genes involved in other QTL analyses.

The completion of the genome sequence brings us several major advantages that we can use in positional cloning [6]. First of all, all of the coding sequences of a chromosomal region of interest have been established. Secondly, information on the introns and the 5′ and 3′ untranslated regions of the genes are available, making it possible for the gene to be analyzed thoroughly throughout the coding region and the regulatory region. Thirdly, the availability of all sequences of a chromosomal region, nucleotide organization, gene ordering, gene expression patterns, and chromosomal structure allow these aspects to be analyzed. At the same time, high throughput technologies for mutation analysis and gene profiling have also been developed rapidly to meet the new needs. All of these developments have improved our search for candidate genes in positional cloning.

Use of these advanced genomic tools combined with our access to unique mouse resources enhanced our ability to identify important QTGs [1], [7]–[9]. Specifically, we were able to take advantage of recombinant inbred (RI) strains derived from C57BL/6J (B6) X DBA/2J (D2), known as BXD, for the identification of causal gene(s) associated with BMD [13]–[15]. The combined BXD strain set is the largest mouse RI mapping panel currently available. All of the BXD strains have now been genotyped at high density (625,000 SNPs). Furthermore, both parental strains have been completely sequenced (DBA/2J by our group at UTHSC using the SOLiD systems (20X mate-pair shotgun reads of 1 kb, 2 kb, and 4 kb libraries- ABI's SOLiD Sequencer). Approximately 1.8 million SNPs have been characterized between two parental strains. This provides unprecedented power in screening candidate genes and can reduce the effective length of QTL intervals. It also makes it possible to reverse standard mapping strategies and to explore downstream effects of known sequence variants.

Previously, as many as 24 recombinant inbred (RI) strains derived from BXD mice were used to detect QTL for BMD in female mice [11]–[12]. In the experiments reported here we used a total of 46 strains; each strain includes both male and female mice at. By using PIXImus and bioinformatics tools, we sought to identify gender-specific QTL for BMD based on separate analysis of both tibia and femur samples [17]–[18]. There was an excellent correlation between both bones. Surprisingly we found that the strongest predictor of bone density was in male mice and was located on chromosome 15. By analysis of the genetic composition of the QTL we were able to identify a candidate gene and to identify a specific mutation responsible for the effect of the QTL. Furthermore, we were able to identify a similar pattern in human samples.

## Materials and Methods

### Animals

Mice from BXD RI strains were obtained from the breeding facilities at the University of Tennessee Health Science Center (UTHSC). All mouse experimental procedures and husbandry were performed in accordance with the National Institutes of Health's Guide for the Care and Use of Laboratory Animals and approved by the UTHSC Institutional Animal Care and Use Committee. Mice were housed in microisolator cages under SPF conditions. They were fed standard rodent chow ad libitum. When the mice were 3 months old they were euthanized and the femurs and tibiae were harvested by sharp dissection to remove muscle and connective tissue and stored in 70% ethanol until analyzed.

### Bone property measurement and other phenotypes

Bone property measurements were conducted using the PIXImus dual-energy X-ray absorptiometer (DXA) (GE Lunar PIXImus, GE Healthcare, WI). Calibration of the instrument was conducted as recommended by the manufacturer. One femur (left) and one tibia (left) from each mouse was used to measure bone mineral content (BMC), bone area (BA), and BMD. All the bones were analyzed in the same orientation on the scan window. All scans were performed by the same person who was unaware of the specific strains from which the bones were obtained. Tibia and femur were normalized for age and sex using generalized linear regression model. The statistical analyses were performed with SAS software version 9.3 (SAS Institute Inc, Cary, NC).

### QTL/interval analysis

QTL mapping was conducted using publically available software on GeneNetwork (http://www.genenetwork.org/webqtl/main.py). One important feature of the GeneNetwork is WebQTL, which is the leading GeneNetwork module, and has been optimized for on-line analysis of traits that are controlled by combinations of allelic variants and environmental factors [10]. A simple graphical user interface enables rapid, intuitive mapping, and analysis of the reconstructed network [16]. For these experiments we entered the BMD as determined by Piximus analysis of both femurs and tibia. For mapping, 2,000 permutation tests were conducted to determine statistical significance. The threshold was computed by evaluating the distribution of highest LRS scores generated by a set of 2,000 random permutations of strain means (http://www.genenetwork.org/glossary.html). The significant threshold by this calculation represents the approximate LRS value that corresponds to a genome-wide p-value of 0.05, or a 5% probability of falsely rejecting the null hypothesis that there is no linkage anywhere in the genome [21]. The suggestive threshold represents the approximate LRS value that corresponds to a genome-wide p-value of 0.63, or a 63% probability of falsely rejecting the null hypothesis that there is no linkage anywhere in the genome [21]–[22]. After we obtained the initial loci of QTL from WebQTL from GeneNetwork [20], we further examined the genomic regions of QTL of significant loci for potential associated genes.

### Analysis for candidate genes within QTL

Using following searching terms—BMD, abnormal bone mineralization, bone mineral density—we identified potential candidate genes for QTL with PGMapper (http://www.genediscovery.org/pgmapper/index.jsp)[19]. Mouse strain-specific single nucleotide polymorphism (SNP) information of potential candidate genes was obtained using NCBI (http://www.ncbi.nlm.nih.gov/projects/SNP/MouseSNP.cgi) and The Jackson Laboratory database (http://www.informatics.jax.org/javawi2/servlet/WIFetch?page=snpQF).

### Genotype analysis of *Trps1*

Genomic sequencing and SNP analysis were done at the Molecular Resource Center, UTHSC. From the mouse SNP database and previous report, we know that at position 50653875 on mouse chromosome 15, there is a C/G change which is in a coding region and causes a V [Val] ⇒ L [Leu] amino acid change (*rs32398060*). We confirmed the existence of the C/G change between B6 and D2 and its segregation among the BXD strains by direct sequencing using a pair of primers: 5′- AAGGAAACCTTCCTACCCGT-3′, 5′- CATACCCACCATCAAAGAAG-3′.

### Human samples

Four samples were incorporated, of which two were from in-house studies (Tulane University) and two were identified from the database of genotype and phenotype (dbGAP). The two in-house samples consisted of 987 (Omaha osteoporosis study, OOS) and 2,250 (Kansas-city osteoporosis study, KCOS) unrelated individuals, respectively, of European ancestry. The third sample was derived from the Framingham Heart Study (FHS), a longitudinal and prospective cohort comprising >16,000 individuals spanning three generations, of European ancestry]. Focusing on the first two generations, we identified 3,747 phenotyped individuals. The fourth sample was the Indiana Fragility Study (IFS), a cross-sectional cohort comprising 1,493 premenopausal sister pairs of European ancestry [23]–[24]. After quality control, 1,488 subjects were qualified for analysis. Among them, female samples are 490, 1,708, 2,147, and 1,488, for OOS, KCOS, FHS, and Indiana, respectively.

### Modeling of human phenotypes

In each of the four samples, covariates were screened among gender, age, age^2^, weight, height, scan side (in FHS) with the step-wise linear regression model. Raw BMD measurements were adjusted by significant covariates. To adjust for potential population stratification, the first five principal components derived from genome-wide genotype data were included as covariates. Residual phenotypes after adjustment were normalized by inverse quantile of the standard normal distribution to impose a normal distribution on phenotypes that were analyzed subsequently.

### Genotyping of human populations

Experiments and analyses of human samples were parallel to an ongoing genome-wide association study in humans and were performed on a genome-wide scale, though results of only SNPs in the *Trps1* gene were used in the present study. All samples were genotyped by high-throughput SNP genotyping arrays (Affymetrix Inc., Santa Clara, CA; or Illumina Inc., San Diego, CA, USA) following the manufacturer's protocols. Quality control of genotype data were implemented with Plink [25], with the following criteria applied: individual missingness<5%, SNP call rate >95%, and Hardy-Weinberg equilibrium (HWE) p-value >1.0×10^−5^. For familial samples (FHS and IFS), all genotypes with the Mendel error were set to missing. Population outliers were monitored by principal components derived from genome-wide genotype analysis.

### Genotype imputation of human populations

Each GWAS sample was imputed by the 1000 genomes project sequence variants (as of August, 2010). Reference haplotypes representing 283 individuals with European ancestry, 193 with Asian ancestry, and 174 with African ancestry were downloaded from MACH] download website. Each GWAS sample was imputed by the respective reference panel with the closest ancestry.

Prior to imputation, a consistency test of allele frequency between GWAS and reference samples was examined with the chi-square test. To correct for potential mis-strandedness, GWAS SNPs that failed a consistency test (p<1.0×10^−6^) were transformed into reverse strand. SNPs that again failed consistency were removed from the GWAS sample.

To distribute imputation computation to multiple parallel CPUs, chromosomes were split into non-overlapping fragments each of 10 Mega base-pair length. In each fragment, haplotypes of individual GWAS were phased by a Markov Chain Haplotyping algorithm (MACH)(26).For familial samples (FHS and IFS), 200 unrelated founder individuals were randomly selected to estimate model parameters, which were then used to impute all family members. Based on phased haplotypes, untyped genotypes were then imputed by a computationally efficient imputing algorithm Minimac [26]. Each GWAS sample was imputed by relevant population's reference haplotypes. SNPs with *r* ^2^ score smaller than 0.3 as estimated by Minimac were considered of poor imputation accuracy. SNPs of high accuracy in at least two samples, and of minor allele frequency (MAF) >0.05 in at least one sample, were included for subsequent analyses [27].

### Association analysis of human BMD and *TRPS1* SNPs

Each GWAS sample was tested for association between phenotypes and genotyped and imputed SNPs under an additive mode of inheritance. For unrelated samples, association was examined by the linear regression model with MACH [28]–[29], in which allele dosage was taken as the predictor for the phenotype. For familial samples (FHS and IFS), a mixed linear model was used in which the effect of genetic relatedness within each pedigree was also taken into account [30]. Genomic control inflation factor was estimated for each individual GWAS. Associations in both the gender combined sample and gender-specific samples were examined.

### Meta-analysis of human populations

Summary statistics of associations from each sample were combined to perform weighted fixed-effect meta-analysis with METAL [31], in which weights were proportional to the square-root of each sample size. *Cochran*'s Q statistic and *I* ^2^ were calculated as measures of between-study heterogeneity [32]. The student t-test and correlation were used in comparison of data between women and men.

## Results

### QTL loci obtained from BXD strains

**Figure 1:**
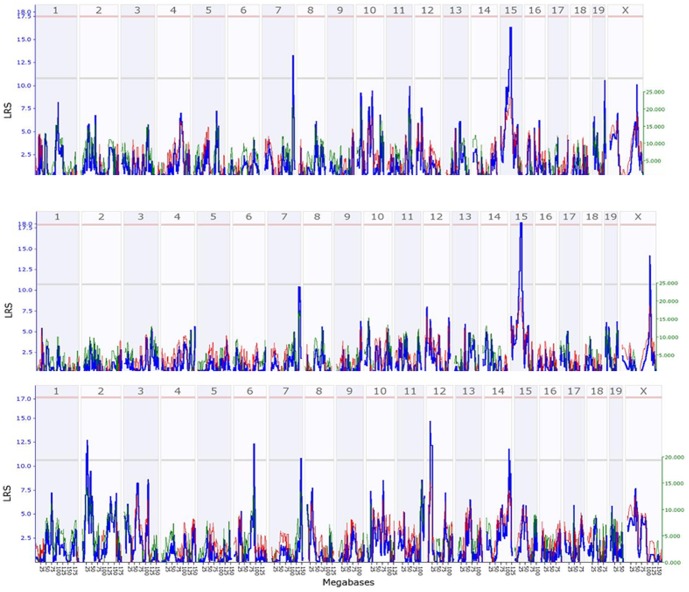
QTL of BMD of the femur and tibia in female and male mice. The numbers on top of each figure indicate the number of chromosome. Numbers on left vertical bar indicate the LRS values. Figure 1A. QTL of BMD detected for femur in male mice. Figure 1B. QTL of BMD detected for tibia in male mice. Figure 1C. QTL of BMD detected for femur in female mice.

From each of the 370 mice from 46 strains, we measured the BMD of a femur and a tibia. Using GeneNetwork mapping, we conducted QTL mapping on BMD of a femur and tibia from both female and male mice. There was a considerable difference in BMD among strains, between the two progenitor strains B6 and D2, and between females and males (Figure S1). For QTL of femurs of male mice, permutation tests indicated the following scores: suggestive likelihood ratio statistic (LRS) = 10.78, significant LRS = 17.96, and highly significant LRS = 21.46 (P≤0.01). A QTL on Chr 15 was suggestive of regulation for BMD with additive effect of as much as -23.508 (Figure 1A) with LRS = 15.731. The peak region of QTL was approximately between 38 Mbp and 52 Mbp. A second QTL locus was detected on Chr7, with LRS of 13.285. The additive effect of the QTL was 20.982 (Figure 1A). For QTL of tibiae BMD of male mice, permutation tests estimated the scores of suggestive LRS = 10.68, significant LRS = 17.64, and highly significant LRS = 21.95. The QTL on Chr 15 reached a significant level with an LRS of more than 18 (Figure 1B). The genomic region of the QTL was the same as in the femur. In addition, a suggestive QTL on Chr X in males had an LRS of 14.183 and additive effect of -18.730. For QTL of femoral BMD of female mice, permutation tests indicated the suggestive LRS = 10.58, significant LRS = 17.73, and highly significant LRS = 20.82. Significant QTL were detected on five chromosomes: 2, 6, 7, 12, and 14 (Figure 1C). For QTL of tibiae BMD of female mice, we did not detect any genomic regions with an LRS greater than the suggestive level (LRS 10.55, based on 2,000 permutation tests) from tibiae of female mice. The top LRS regions, however, were located on Chr 3, 11, and 12 (data not shown).

### QTL on mouse Chr 15 for the male mouse

The QTL on Chr 15 for the BMD in the femur and tibia in male mice was mapped in the same location and dominated QTLs on all other chromosomes. The QTLs from the femur and tibia were mapped in the same genomic region. DBA/2J alleles reduced BMD on average by approximately 5%. Most importantly, B6 genotype had a positive impact, while the D2 genotype had a negative impact on BMD for males. Furthermore, the region was relatively small, considering the usual size of a QTL. It was located between 38 Mbp and 52 Mbp, (peak region with LRS of 15, data not show). Within this region, there are 73 genes/transcripts, (Table S1). By searching those transcripts with PGMapper [19], we found one important candidate gene: zinc finger transcription factor for trichorhinophalangeal syndrome type I protein (*Trps1*). *Trps1* has been reported by Churchill and colleagues [18] as the probable candidate gene for the QTL of BMD in an SM/J by NZB/BlNJ intercross population and recently for hip geometry in humans as well as BMD in mice by Ackert-Bicknell and colleagues at the Jackson Laboratory[33].

### Correlation between genotype of *Trps1* and BMD phenotype of the femur and tibia in male mice

**Figure 2:**
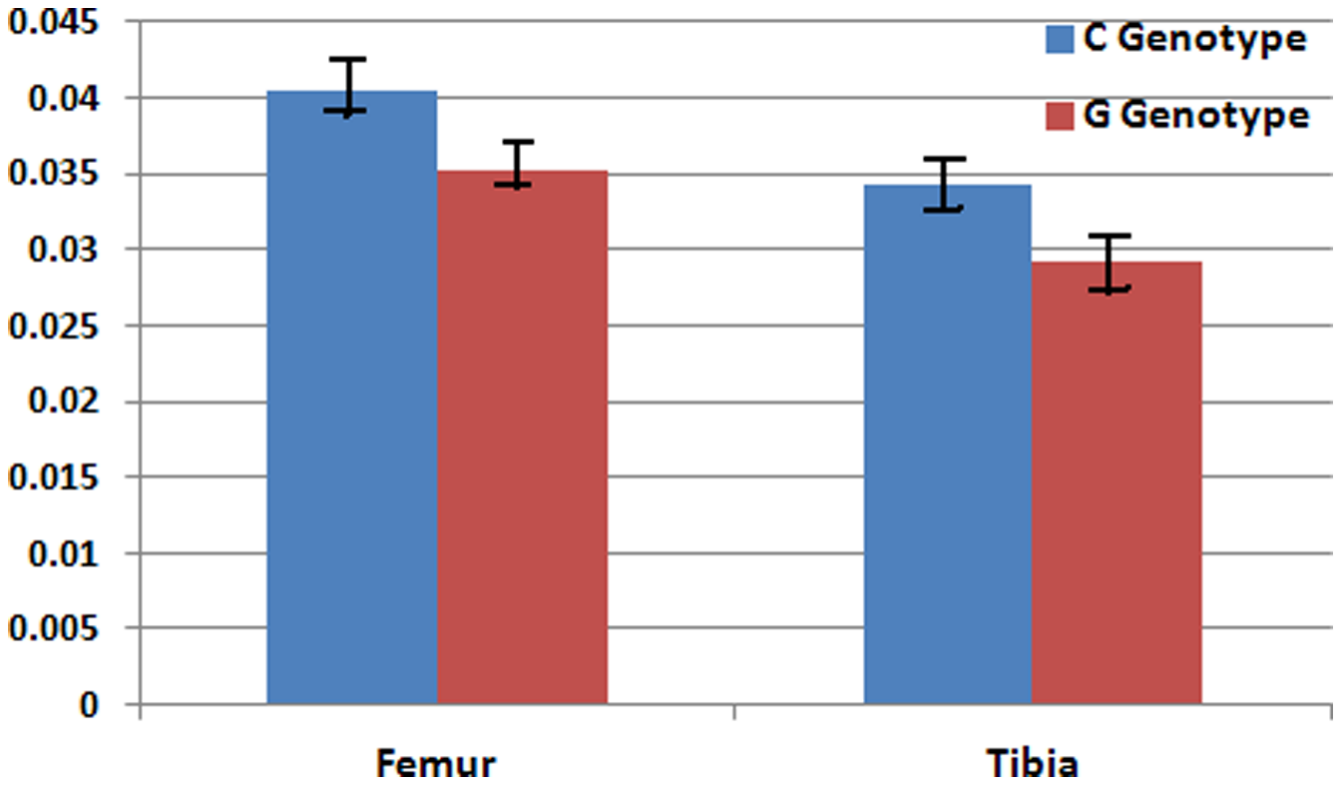
*Trps1* as the candidate gene for BMD in male. BMD of “G” and “C” genotypes of femur and tibia in male mice.

We next examined the SNPs of *Trps1* including 2 kb up- and downstream between C57BL/6J and DBA/2J by using MGI http://www.informatics.jax.org/javawi2/servlet/WIFetch?page=snpQF. Interestingly, *Trps1* is in a highly polymorphic genomic region. The region between 50,485,541 bp and 50,722,748 bp has 392 SNPs. Among those SNPs, 182 are different between C57BL/6J and DBA/2J. At position of 50,653,875 bp, change (G/C) in the coding region causes a V to L change in the amino acid sequence. According to the Ensembl database (http://useast.ensembl.org/Mus_musculus/Location/View?g=ENSMUSG00000038679r=15:50486307-50721587), *Trps1* has six transcripts, all coding for protein; four transcripts, *Trps1*-201, *Trps1*-202, *Trps1*-203, and *Trps1*-205, contain seven exons. The other transcripts contain fewer exons or none at all. The C/G change at position of 50653875bp of *Trps1* is present in exon 5, which is in five of the six transcripts. Previous investigations [12], [33] have indicated that the change from valine to leucine does not change polarity or the acid-base properties, but it does seem to be conserved across species and lies in a conserved region of the protein. Because this variation was not listed in the genotype of BXD strains, we sequenced the polymorphic site in *Trps1* in 39 BXD RI strains (figure S2). We then compared the BMD of C and G genotypes. There was a highly significant difference in BMD in both the femur (*P* = 0.0086) and tibia (*P* = 0.0005) between the C and G genotypes in male mice (Figure 2). In female mice, the difference in the femur or tibia was not significant (*P* = 0.2098 and 0.4502).

### Verification of sex effect on QTL detection

**Figure 3:**
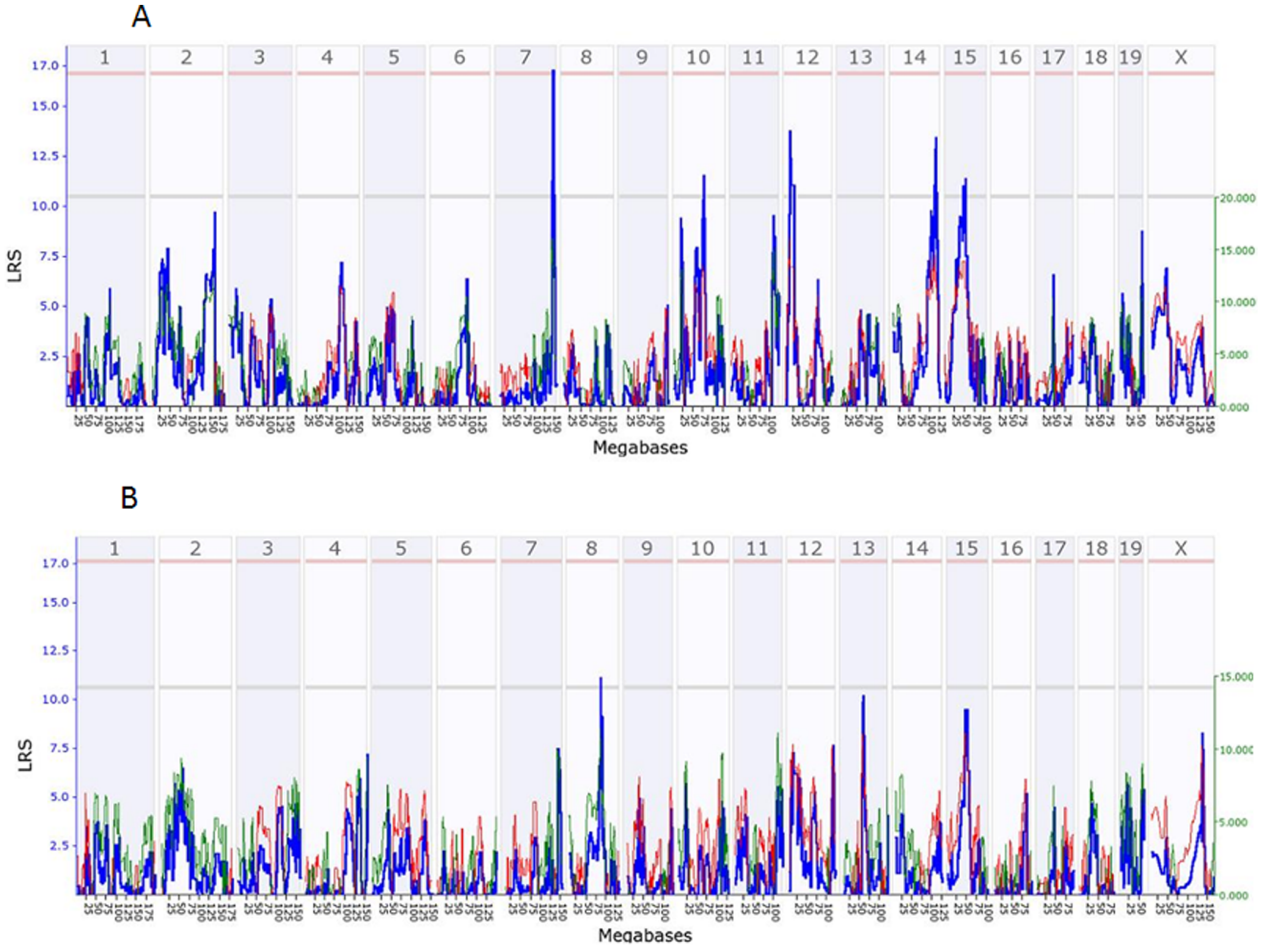
QTL on chromosome 15 detected from both sexes and from female mice. Interval mapping was conducted using age- and sex-adjusted BMD data and data of the femur of female mice. Figure 3A. QTL for BMD detected from data of mouse femur. Figure 3B. QTL for BMD detected from data of mouse tibia.

Previously two studies using mouse models have reported *Trps1* as the candidate gene for BMD. Ishimori et al.[18] identified QTL for BMD in an SM/J by NZB/BlNJ intercross (both sexes) population and identified *Trps1* as a probable candidate gene for a QTL that explains the smallest percentage of variance (2.2%) of vertebral BMD among a total of 9 QTLs. The other report is from Ackert-Bicknell et al [33]. These authors used the data from female mice, however, with a bi-allelic method with genotype data for only Chr 15. In order to test the sex effect on QTL, we performed the interval mapping with age- and sex-adjusted BMD data. As shown in Figure 3, in both the femur and tibia, a peak was identified on Chr15 (Figure 3A, 3B). In the femur, the QTLs with higher LRS scores were on chromosomes 7, 10, 12, and 14, while the QTLs on Chr15 also reached the suggestive level. In the tibia, only a QTL on Chr 8 reached a suggestive level, while a peak on Chr15 did not reach a suggestive level. Thus, the data from both sexes neutralized the sex effect of QTL on Chr 15 to a much lower level. These data confirm the sex-specific influence of QTL on Chr 15.

### Gender difference of *TRPS 1* genotypes and BMD in human population

**Figure 4:**
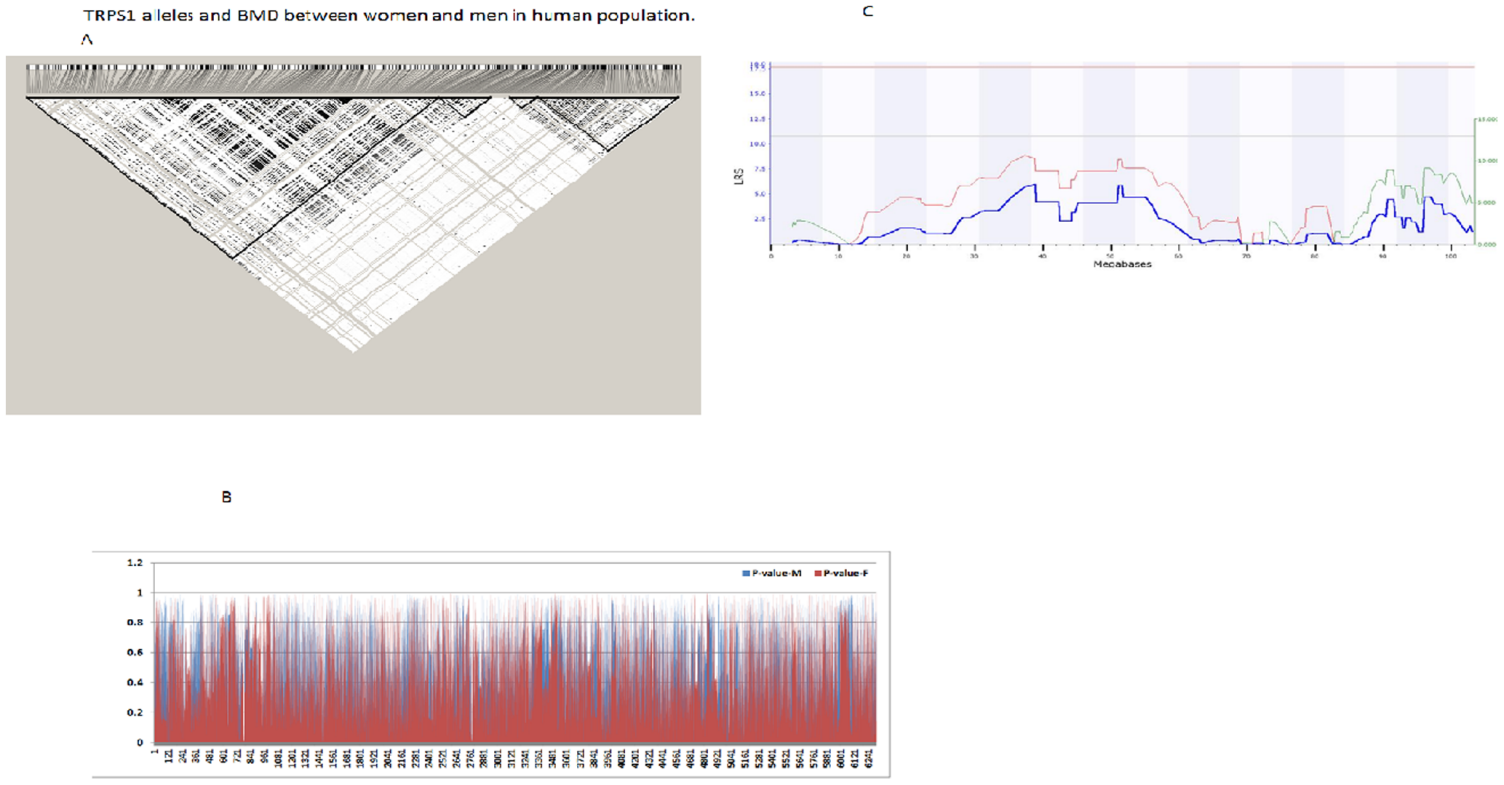
TRPS1 alleles and BMD between women and men in human population. Figure 4A. The linkage disequilibrium (LD) plot of the SNPs within the TRPS1 gene. It was plotted by the software Haploview. Figure 4B. P values of 6310 among women and men. The lower the P value is, the stronger association between SNP allele and BMD is. Y-bar indicates the P values of each SNP in women and in men with different colors. X-bar indicates the number of SNP. Different color in many individual bars can be seen because the different P values between women and men in those SNP alleles. Figure 4C. Potential detection of QTL using data of BMD of the femur in female mice.

We analyzed the allele frequency and distribution of 6,310 SNPs among 5,833 women and 2,639 men of European ancestry. For each SNP, we obtained the frequency of alleles, minimal and maximal frequency of alleles, and P value. We then separated the data of women and men and compared those parameters between the two groups. The frequency of alleles and the minimal and maximal frequency of alleles were highly positively correlated between genders. Particularly, P value of the allele frequency between women and men was 0.9988 and R value was 0.9999. The R value of minimal and maximal frequency between women and men were 0.9998 and 0.9998, respectively. Figure 4A LD plots with the haplotype structure of the TSP1 locus. The data indicate that all alleles are similarly distributed between women and men. We then tested the association/correlation between each of the 6,310 SNPs and BMDs. A positive association indicates that the increase in dosage of a particular allele at that SNP will cause a change in BMD value. In our case, the positive/negative conclusion was driven by a statistical p-value: the smaller the p-value, the stronger the evidence of positive association. We next compared the P values of all alleles between women and men, as P values represent the degree of association of an allele and BMD (Pp  =  T test of P values, Rp  =  correlation of P values of 6310 SNP between 5833 women and 2639 men). Similar to the results obtained in the BXD studies, we observed that there was a significant difference in associations among SNP alleles and BMD between women and men (Figure 4B). Because of the differences among P values, two colors of bars can be seen. The overall Pp value between P-values of women and men is 2.18×10^−13^. Analysis of correlations indicated that there is no correlation of P values between women and men, with Rp = 0.075.

## Conclusion and Discussion

Our data clarified the major function of the *Trps1* as a sex-specific regulator of BMD of the femur and tibia in mice. Previously two studies using mouse models have reported *Trps1* as the candidate gene for BMD. Ishimori et al.[18] identified QTL for BMD in an SM/J by NZB/BlNJ intercross (both sexes) population and identified *Trps1* as a probable candidate gene for a QTL that explains the smallest percentage of variance (2.2%) of vertebral BMD among a total of 9 QTLs. The other report, from Ackert-Bicknell et al [33], used data from female mice with a bi-allelic method with genotype data for only Chr 15. The advantage of our study was the comparison of RI strains of female and male mice separately. In previous studies, most QTL mapping has been done with female mice.[33], [34]–[35]. The number of RI strains in male mice in our study represents the largest population in one RI strain so far in the study of BMD. *Trps1* has been identified as a candidate gene of BMD [18], [33]. Those studies provided significant knowledge in the functional study of *Trps1* in skeletal development. The sex specificity of *Trps1* has not been known, however, partially because of the lack of large number of males in the animal models. It is known that pair-scan procedures need careful diagnostics and it is very sensitive to outliers. In our analysis, we conducted interval mapping with 2000 permutations. The other advantage in our analysis is that, unlike the F2 population, we had multiple mice in each strain. The BXD strains also have been used for the study of BMD. The first study using 24 BXD strains was done in female mice [11]. The second study used a relatively small number of RI strains [12]. Our study benefits from the large number of strains and separate analysis of both sexes.

For the first time, our data reveal the *Trps1* as the most likely primary causal gene of BMD in the sex-specific QTL region on Chr 15. The large amount of genomic resources accumulated in recent years greatly enhanced our analysis. Our study took advantage of density mapping of BXD RI strains and the small peak region of the QTL. Unlike most QTL mappings, which locate the causal gene(s) in a large chromosomal region with hundreds of genes, our QTL had a peak region of 12 transcripts. Our haploid analysis further narrowed the region into 8 transcripts with only two known genes. SNP and expression analysis then ruled out the possibility of other candidates, leaving *Trps1* as the only most likely candidate.

Our data do not rule out the function of *Trps1* in female bone development. In the interval analysis for BMD of the femur in female mice, we found a peak on Chr 15 (Figure 4C), although it did not reach the significant level. We believe that, with a large population, it is possible that the QTL on Chr 15 in females might reach a suggestive or a significant level. Klein et al. identified the QTL on Chr15 with a small effect on the whole body BMD using 24 BXD RI strains [11]. Beamer and others detected a QTL of BMD on the other regions of Chr 15 [34]–[35]. Our data agree with the previous studies that identified major QTLs on Chr 1, 4, 11 and small effect QTL on Chr 15 [11], [34]–[35] using F2 population of female mice.

In humans, TRPS1 mutation causes the trichorhinophalangeal syndrome, which generally includes skeletal dysplasia characterized by a triad of hair, craniofacial, and skeletal abnormalities [36]–[37]. However, there is variability in the clinical findings with considerable overlap, even among subjects of the same family[38]–[41]. In addition to the large deletion and alteration of chromosomes, most missense mutations are from exon 6. Thus, the effect on mutations in other parts of the *Trps1* gene, which in many cases do not result in trichorhinophalangeal syndrome, have not been given much attention. Those mild mutations, on the other hand, may result in a mild effect such as osteoporosis.

One possible limitation of our study is that we measured the BMD with the Lunar PIXImus Densitometer (GE Healthcare, Fairfield CT) and used mice 3 months of age. The two previous reports on *Trps1* in mice were done with mice at older ages. Despite limitations in the software, PIXImus is a valuable tool for studying BMD and skeletal development of small animals [11]–[12]. Although the measurements obtained from PIXImus generally correlate with those obtained from microCT, they are not always identical [42].

In summary, using a combination of a large number of RI strains and genomic resources, we identified Trps1 as the most likely causal gene for the sex-specific QTL on mouse Chr15. Data from a human population also suggested a gender difference in the association of BMD and TRPS1 SNP.

## Supporting Information

Figure S1 **Average mouse BMD among BXD RI strains, F1s, and the two progenitor strains B6 and D2.** Figure s1A, BMD of femurs in female; Figure s1B, BMD of femurs in male; Figure s1C, BMD of Tibia in Female; Figure s1D, BMD of Tibia in male.
(DOC)

Figure S2 **Polymorphic site in** ***Trps1*** **between B6 and D2 strains.** Arrows point to the polymorphic site.
(TIF)

Table S1 **Candidate genes within QTL region on mouse chromosome 15.**
(DOCX)

## References

[1] Xiong Q, Jiao Y, Hasty KA, Canale ST, Stuart JM, et al (2009) Quantitative trait loci, genes, and polymorphisms that regulate bone mineral density in mouse. Genomics 93(5): 401–414 10.1016/j.ygeno.2008.12.008. Epub 2009 Jan 14 19150398 PMC2901167

[2] Garnero P, Delmas PD (2004) Contribution of bone mineral density and bone turnover markers to the estimation of risk of osteoporotic fracture in postmenopausal women. J Musculoskelet Neuronal Interact 4(1): :50–63. Review. 15615078

[3] Tremollieres F, Ribot C (2010) Bone mineral density and prediction of non-osteoporotic disease. Maturitas 65(4): 348–351 10.1016/j.maturitas.2009.12.023. Epub 2010 Jan 15 20079983

[4] Bonjour JP, Theintz G, Law F, Slosman D, Rizzoli R (1994) Peak bone mass. Osteoporos Int 4 Suppl 17–13. 8081064 10.1007/BF01623429

[5] Schnitzler CM (1993) Bone quality: a determinant for certain risk factors for bone fragility. Calcif Tissue Int 53 Suppl 1S27–31. 8275376 10.1007/BF01673398

[6] Jiao Y, Jin X, Yan J, Zhang C, Jiao F, et al (2008) A deletion mutation in Slc12a6 is associated with neuromuscular disease in gaxp mice. Genomics 91(5): 407–414 10.1016/j.ygeno.2007.12.010. Epub 2008 Mar 14 18343091 PMC2430873

[7] Milner LC, Buck KJ (2010) Identifying quantitative trait loci (QTLs) and genes (QTGs) for alcohol-related phenotypes in mice. Int Rev Neurobiol 91: 173–204 10.1016/S0074-7742(10)91006-4. Review 20813243

[8] Li GH, Cheung CL, Xiao SM, Lau KS, Gao Y, et al (2011) Identification of QTL genes for BMD variation using both linkage and gene-based association approaches. Hum Genet 130(4): 539–546 10.1007/s00439-011-0972-2. Epub 2011 Mar 19 21424381 PMC3178777

[9] Grubb SC, Churchill GA, Bogue MA (2004) A collaborative database of inbred mouse strain characteristics. Bioinformatics 20(16):2857–2859. Epub 2004 May 6. 10.1093/bioinformatics/bth299 15130929

[10] Peirce JL, Lu L, Gu J, Silver LM, Williams RW (2004) A new set of BXD recombinant inbred lines from advanced intercross populations in mice. BMC Genet 29 5: 7. 10.1186/1471-2156-5-7 PMC420238 15117419

[11] Klein RF, Mitchell SR, Phillips TJ, Belknap JK, Orwoll ES (1998) Quantitative trait loci affecting peak bone mineral density in mice. J Bone Miner Res 13: 1648–1656. 9797472 10.1359/jbmr.1998.13.11.1648

[12] Orwoll ES, Belknap JK, Klein RF (2001) Gender specificity in the genetic determinants of peak bone mass. J Bone Miner Res 16: 1962–1971. 11697792 10.1359/jbmr.2001.16.11.1962

[13] Andreux PA, Williams EG, Koutnikova H, Houtkooper RH, Champy MF, et al (2012) Systems genetics of metabolism: the use of the BXD murine reference panel for multiscalar integration of traits. Cell 150(6): 1287–1299 10.1016/j.cell.2012.08.012. Epub 2012 Aug 30 22939713 PMC3604687

[14] Suwanwela J, Farber CR, Haung B, Song B, Pan C, et al (2011) Systems genetics analysis of mouse chondrocyte differentiation. Journal of Bone and Mineral Research 26: 746–760. 10.1002/jbmr.271 PMC3179327 20954177

[15] Lynch RM, Naswa S, Rogers Jr GL, Kanla SA, Das S, et al (2010) Identifying genetic loci and spleen gene coexpression networks underlying immunophenotypes in the BXD recombinant inbred mice. Physiological Genomics 41: 244–253. 20179155 10.1152/physiolgenomics.00020.2010 PMC4073992

[16] Chesler EJ, Lu L, Shou S, Qu Y, Gu J, et al (2005) Genetic dissection of gene expression reveals polygenic and pleiotropic networks modulating brain structure and function. Nature Genetics 37: 233–242. 15711545 10.1038/ng1518

[17] Soon G, Quintin A, Scalfo F, Antille N, Williamson G, et al.. (2006) PIXImus bone densitometer and associated technical measurement issues of skeletal growth in the young rat. Calcif Tissue Int 78:186–192. Epub 2006 Mar 17. 10.1007/s00223-005-0191-8 16547639

[18] Ishimori N, Stylianou IM, Korstanje R, Marion MA, Li R, et al (2008) Quantitative trait loci for BMD in an SM/J by NZB/BlNJ intercross population and identification of *Trps1* as a probable candidate gene. J Bone Miner Res 23: 1529–1537. 18442308 10.1359/JBMR.080414 PMC2586053

[19] Xiong Q, Qiu Y, Gu W (2008) PGMapper: a web-based tool linking phenotype to genes. Bioinformatics 24: 1011–1013. 18204061 10.1093/bioinformatics/btn002 PMC2505180

[20] Geisert EE, Lu L, Freeman-Anderson NE, Templeton JP, Nassr M, et al (2009) Gene expression in the mouse eye: an online resource for genetics using 103 strains of mice. Mol Vis 15: 1730–63. 19727342 PMC2736153

[21] Wang J, Williams RW, Manly KF (2003) WebQTL: web-based complex trait analysis Wang J, Williams RW, Manly KF. Neuroinformatics. 1(4): 299–308. 10.1385/NI:1:4:299 15043217

[22] Calabrese G, Bennett BJ, Orozco L, Kang HM, Eskin E, et al.. (2012) Systems genetic analysis of osteoblast-lineage cells. PLoS Genet 8(12):e1003150. PMID: 23300464. 10.1371/journal.pgen.1003150 PMC3531492 23300464

[23] Rivadeneira F, Styrkarsdottir U, Estrada K, Halldorsson BV, Hsu YH, et al (2009) Twenty bone-mineral-density loci identified by large-scale meta-analysis of genome-wide association studies. Nat Genet 41: 1199–206. 19801982 10.1038/ng.446 PMC2783489

[24] Estrada K, Styrkarsdottir U, Evangelou E, Hsu YH, Duncan EL, Ntzani EE, et al (2012) Genome-wide meta-analysis identifies 56 bone mineral density loci and reveals 14 loci associated with risk of fracture. Nat Genet 44: 491–501. 22504420 10.1038/ng.2249 PMC3338864

[25] Purcell S, Neale B, Todd-Brown K, Thomas L, Ferreira MA, et al (2007) PLINK: a tool set for whole-genome association and population-based linkage analyses. Am J Hum Genet 81: 559–575. 17701901 10.1086/519795 PMC1950838

[26] Liu EY, Li M, Wang W, Li Y (2013) MaCH-admix: genotype imputation for admixed populations. Genet Epidemiol 37: 25–37. 23074066 10.1002/gepi.21690 PMC3524415

[27] Pei YF, Zhang L, Liu Y, Li J, Shen H, et al.. (2013) Meta-analysis of genome-wide association data identifies novel susceptibility loci for obesity. Hum Mol Genet. PMID: 24064335. 10.1093/hmg/ddt464 PMC3888264 24064335

[28] Li Y, Willer CJ, Ding J, Scheet P, Abecasis GR (2010) MaCH: using sequence and genotype data to estimate haplotypes and unobserved genotypes. Genet Epidemiol 34: 816–34. 21058334 10.1002/gepi.20533 PMC3175618

[29] Zhang L, Li J, Pei YF, Liu Y, Deng HW (2009) Tests of association for quantitative traits in nuclear families using principal components to correct for population stratification. Ann. Hum Genet 73: 601–13. 10.1111/j.1469-1809.2009.00539.x PMC2764806 19702646

[30] Devlin B, Roeder K (1999) Genomic control for association studies. Biometrics 55: 997–1004. 11315092 10.1111/j.0006-341x.1999.00997.x

[31] Willer CJ, Li Y, Abecasis GR (2010) METAL: fast and efficient meta-analysis of genomewide association scans. Bioinformatics 26: 2190–1. 20616382 10.1093/bioinformatics/btq340 PMC2922887

[32] Higgins JP, Thompson SG, Deeks JJ, Altman DG (2003) Measuring inconsistency in meta-analyses. BMJ 327: 557–60. 12958120 10.1136/bmj.327.7414.557 PMC192859

[33] Ackert-Bicknell CL, Demissie S, Tsaih SW, Beamera WG, Cupples LA, et al.. (2012) Genetic variation in TRPS1 may regulate hip geometry as well as bone mineral density. Bone [Epub ahead of print] 10.1016/j.bone.2012.01.011 PMC3322322 22306695

[34] Beamer WG, Shultz KL, Churchill GA, Frankel WN, Baylink DJ, et al (1999) Quantitative trait loci for bone density in C57BL/6J and CAST/EiJ inbred mice. Mamm Genome 10: 1043–1049. 10556421 10.1007/s003359901159

[35] Yu H, Mohan S, Edderkaoui B, Masinde GL, Davidson HM, et al.. (2007) Detecting novel bone density and bone size quantitative trait loci using a cross of MRL/MpJ and CAST/EiJ inbred mice. Calcif Tissue Int 80:103–110. Epub 2007 Feb 2. 10.1007/s00223-006-0187-z 17308992

[36] Noltorp S, Kristoffersson UL, Mandahl N, Stigsson L, Svensson B, et al (1986) Trichorhinophalangeal syndrome type I: symptoms and signs, radiology and genetics. Ann Rheum Dis 45: 31–36. 3954456 10.1136/ard.45.1.31 PMC1001811

[37] Carrington PR, Chen H, Altick JA (1994) Trichorhinophalangeal syndrome, type I. J Am Acad Dermatol. 31: 331–336. 10.1016/s0190-9622(94)70166-0 8034799

[38] Sarafoglou K, Moassesfar S, Miller BS (2010) Improved growth and bone mineral density in type I trichorhinophalangeal syndrome in response to growth hormone therapy. Clin Genet 78: 591–593 10.1111/j.1399-0004.2010.01434.x 20569260

[39] Tariq M, Ahmad S, Ahmad W (2008) A novel missense mutation in the TRPS1 gene underlies trichorhinophalangeal syndrome type III. Br J Dermatol. 159:476–478. Epub 2008 Jun 9. 10.1111/j.1365-2133.2008.08658.x 18544079

[40] Rossi A, Devirgiliis V, Panasiti V, Borroni RG, Carlesimo M, et al.. (2007) Missense mutation in exon 7 of TRPS1 gene in an Italian family with a mild form of trichorhinophalangeal syndrome type I. Br J Dermatol 157:1021–1024. Epub 2007 Sep 13. 10.1111/j.1365-2133.2007.08158.x 17854380

[41] Vaccaro M, Guarneri F, Barbuzza O, Gaeta M, Guarneri C (2009) A familial case of trichorhinophalangeal syndrome type I. Pediatr Dermatol 26: 171–175. 19419465 10.1111/j.1525-1470.2009.00905.x

[42] Kolta S, De Vernejoul MC, Meneton P, Fechtenbaum J, Roux C (2003) Bone mineral measurements in mice: comparison of two devices. J Clin Densitom 6(3): 251–258. 14514995 10.1385/jcd:6:3:251

