# Supplementary material for: Trps1 Differentially Modulates the Bone Mineral Density between Male and Female Mice and Its Polymorphism Associates with BMD Differently between Women and Men
Source: PLoS One. 2014 Jan 8;9(1):e84485. doi: 10.1371/journal.pone.0084485 (PMC3885592; doi:10.1371/journal.pone.0084485)

**Supplementary Figure S1A. BMD of femurs in Female**


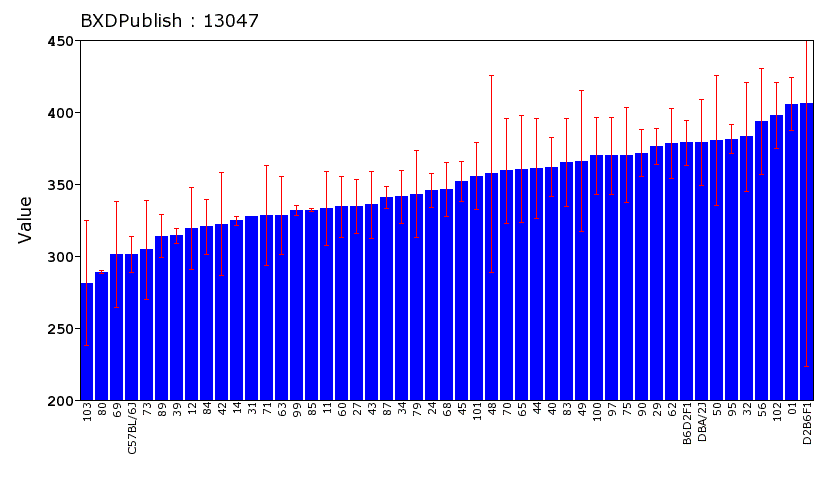


**Figure S1B. BMD of femurs in Male**


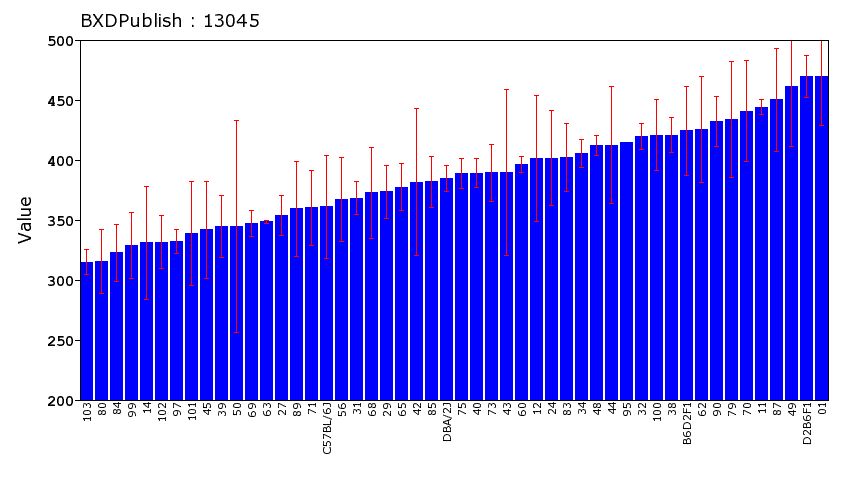


**Figure S1C. BMD of Tibia in Female**


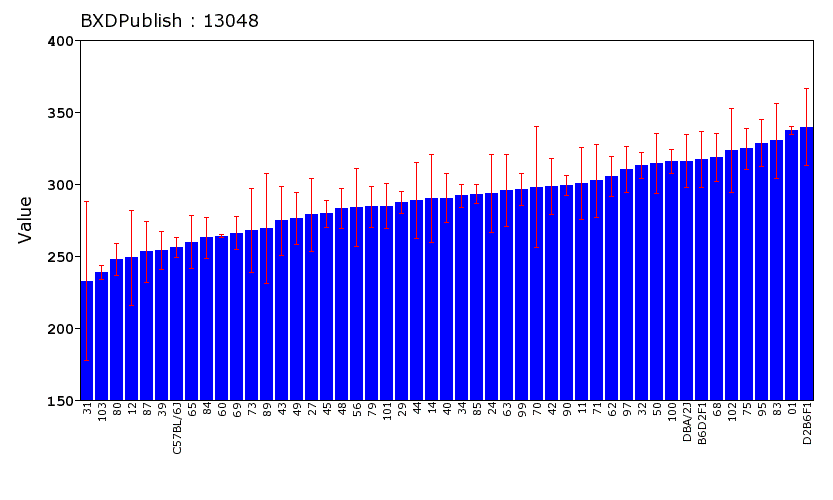


**Figure S1D. BMD of Tibia in Male**


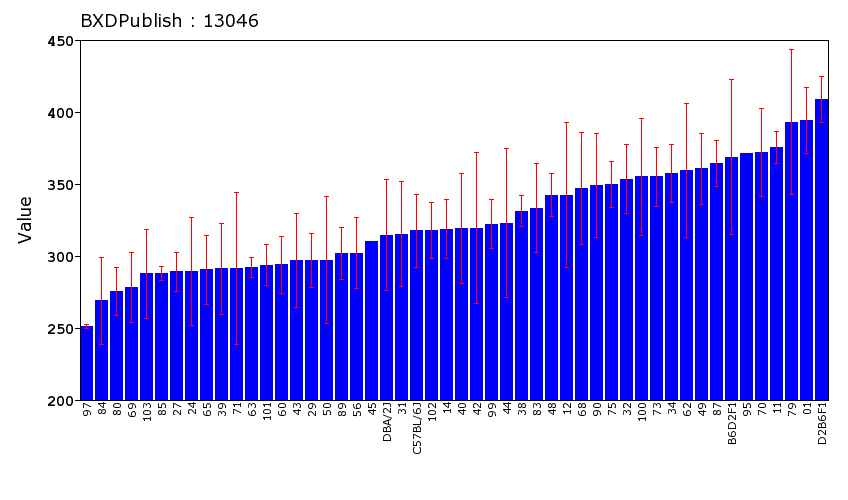

Supplement: Figure S1 — Average mouse BMD among BXD RI strains, F1s, and the two progenitor strains B6 and D2. Figure s1A, BMD of femurs in female; Figure s1B, BMD of femurs in male; Figure s1C, BMD of Tibia in Female; Figure s1D, BMD of Tibia in male. (DOC) [file pone.0084485.s001.doc]
