# Supplementary figures and images for: Trps1 Differentially Modulates the Bone Mineral Density between Male and Female Mice and Its Polymorphism Associates with BMD Differently between Women and Men
Source: PLoS One. 2014 Jan 8;9(1):e84485. doi: 10.1371/journal.pone.0084485 (PMC3885592; doi:10.1371/journal.pone.0084485)

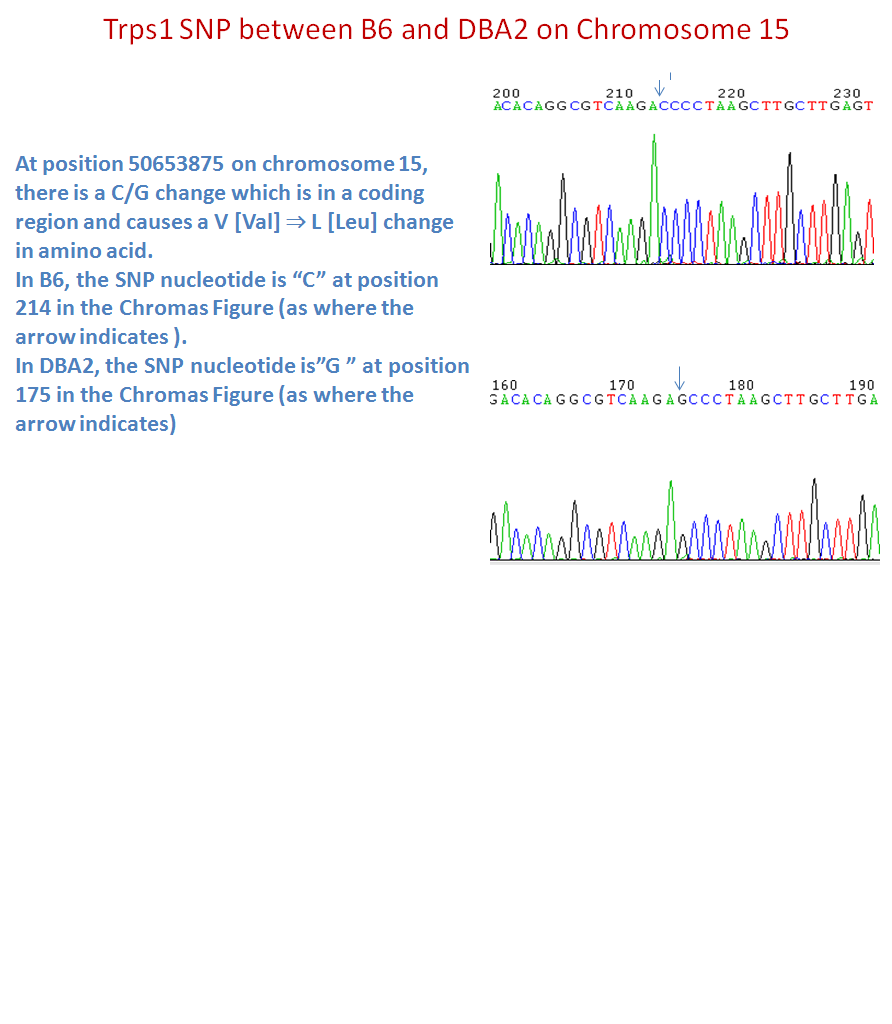

Supplement: Figure S2 — Polymorphic site in Trps1 between B6 and D2 strains. Arrows point to the polymorphic site. (TIF) [file pone.0084485.s002.tif]
