# Supplementary material for: Trps1 Differentially Modulates the Bone Mineral Density between Male and Female Mice and Its Polymorphism Associates with BMD Differently between Women and Men
Source: PLoS One. 2014 Jan 8;9(1):e84485. doi: 10.1371/journal.pone.0084485 (PMC3885592; doi:10.1371/journal.pone.0084485)
Supplement: Table S1 — Candidate genes within QTL region on mouse chromosome 15. (DOCX) [file pone.0084485.s003.docx]

Supplementary Table S1. Candidate genes within QTL region on mouse chromosome 15.

|  | Ensembl Accession | Symbol | Status | Description |
| --- | --- | --- | --- | --- |
| 1 | ENSMUSG00000065852 | SNORA2 | NOVEL | Small nucleolar RNA SNORA2/SNORA34 family [Source:RFAM;Acc:RF00410] |
| 2 | ENSMUSG00000061923 | Odf1 | KNOWN | outer dense fiber of sperm tails 1 [Source:MGI Symbol;Acc:MGI:97424] |
| 3 | ENSMUSG00000096567 | AC122459.1 | NOVEL | NA |
| 4 | ENSMUSG00000097376 | AC122459.2 | NOVEL | NA |
| 5 | ENSMUSG00000037465 | Klf10 | KNOWN | Kruppel-like factor 10 [Source:MGI Symbol;Acc:MGI:1101353] |
| 6 | ENSMUSG00000097645 | AC122397.1 | KNOWN | NA |
| 7 | ENSMUSG00000037458 | Azin1 | KNOWN | antizyme inhibitor 1 [Source:MGI Symbol;Acc:MGI:1859169] |
| 8 | ENSMUSG00000022295 | Atp6v1c1 | KNOWN | ATPase, H+ transporting, lysosomal V1 subunit C1 [Source:MGI Symbol;Acc:MGI:1913585] |
| 9 | ENSMUSG00000097875 | AC164883.1 | NOVEL | NA |
| 10 | ENSMUSG00000022296 | Baalc | KNOWN | brain and acute leukemia, cytoplasmic [Source:MGI Symbol;Acc:MGI:1928704] |
| 11 | ENSMUSG00000022297 | Fzd6 | KNOWN | frizzled homolog 6 (Drosophila) [Source:MGI Symbol;Acc:MGI:108474] |
| 12 | ENSMUSG00000077470 | SNORA17 | KNOWN | Small nucleolar RNA SNORA17 [Source:RFAM;Acc:RF00560] |
| 13 | ENSMUSG00000054196 | Cthrc1 | KNOWN | collagen triple helix repeat containing 1 [Source:MGI Symbol;Acc:MGI:1915838] |
| 14 | ENSMUSG00000022299 | Slc25a32 | KNOWN | solute carrier family 25, member 32 [Source:MGI Symbol;Acc:MGI:1917156] |
| 15 | ENSMUSG00000022300 | Dcaf13 | KNOWN | DDB1 and CUL4 associated factor 13 [Source:MGI Symbol;Acc:MGI:2684929] |
| 16 | ENSMUSG00000084562 | n-R5s39 | KNOWN | nuclear encoded rRNA 5S 39 [Source:MGI Symbol;Acc:MGI:4421884] |
| 17 | ENSMUSG00000037386 | Rims2 | KNOWN | regulating synaptic membrane exocytosis 2 [Source:MGI Symbol;Acc:MGI:2152972] |
| 18 | ENSMUSG00000095297 | AC101886.1 | NOVEL | NA |
| 19 | ENSMUSG00000022303 | Dcstamp | KNOWN | dentrocyte expressed seven transmembrane protein [Source:MGI Symbol;Acc:MGI:1923016] |
| 20 | ENSMUSG00000022304 | Dpys | KNOWN | dihydropyrimidinase [Source:MGI Symbol;Acc:MGI:1928679] |
| 21 | ENSMUSG00000087695 | Gm16291 | NOVEL | predicted gene 16291 [Source:MGI Symbol;Acc:MGI:3826548] |
| 22 | ENSMUSG00000022305 | Lrp12 | KNOWN | low density lipoprotein-related protein 12 [Source:MGI Symbol;Acc:MGI:2443132] |
| 23 | ENSMUSG00000094112 | 9330182O14Rik | KNOWN | RIKEN cDNA 9330182O14 gene [Source:MGI Symbol;Acc:MGI:3045378] |
| 24 | ENSMUSG00000092855 | AC152394.1 | NOVEL | NA |
| 25 | ENSMUSG00000056332 | Gm16294 | KNOWN | predicted gene 16294 [Source:MGI Symbol;Acc:MGI:3645121] |
| 26 | ENSMUSG00000022306 | Zfpm2 | KNOWN | zinc finger protein, multitype 2 [Source:MGI Symbol;Acc:MGI:1334444] |
| 27 | ENSMUSG00000048559 | 4930555K19Rik | KNOWN | RIKEN cDNA 4930555K19 gene [Source:MGI Symbol;Acc:MGI:1922612] |
| 28 | ENSMUSG00000022307 | Oxr1 | KNOWN | oxidation resistance 1 [Source:MGI Symbol;Acc:MGI:2179326] |
| 29 | ENSMUSG00000097232 | AC129212.1 | KNOWN | NA |
| 30 | ENSMUSG00000042895 | Abra | KNOWN | actin-binding Rho activating protein [Source:MGI Symbol;Acc:MGI:2444891] |
| 31 | ENSMUSG00000088469 | SNORA17 | NOVEL | Small nucleolar RNA SNORA17 [Source:RFAM;Acc:RF00560] |
| 32 | ENSMUSG00000022309 | Angpt1 | KNOWN | angiopoietin 1 [Source:MGI Symbol;Acc:MGI:108448] |
| 33 | ENSMUSG00000097805 | AC100734.1 | KNOWN | NA |
| 34 | ENSMUSG00000051920 | Rspo2 | KNOWN | R-spondin 2 homolog (Xenopus laevis) [Source:MGI Symbol;Acc:MGI:1922667] |
| 35 | ENSMUSG00000087905 | 7SK | NOVEL | 7SK RNA [Source:RFAM;Acc:RF00100] |
| 36 | ENSMUSG00000022336 | Eif3e | KNOWN | eukaryotic translation initiation factor 3, subunit E [Source:MGI Symbol;Acc:MGI:99257] |
| 37 | ENSMUSG00000072592 | Gm10373 | KNOWN | predicted gene 10373 [Source:MGI Symbol;Acc:MGI:3642805] |
| 38 | ENSMUSG00000022337 | Emc2 | KNOWN | ER membrane protein complex subunit 2 [Source:MGI Symbol;Acc:MGI:1913986] |
| 39 | ENSMUSG00000088831 | U7 | NOVEL | U7 small nuclear RNA [Source:RFAM;Acc:RF00066] |
| 40 | ENSMUSG00000054409 | Tmem74 | KNOWN | transmembrane protein 74 [Source:MGI Symbol;Acc:MGI:2443417] |
| 41 | ENSMUSG00000038760 | Trhr | KNOWN | thyrotropin releasing hormone receptor [Source:MGI Symbol;Acc:MGI:98824] |
| 42 | ENSMUSG00000088756 | SNORA17 | NOVEL | Small nucleolar RNA SNORA17 [Source:RFAM;Acc:RF00560] |
| 43 | ENSMUSG00000077181 | SNORA17 | NOVEL | Small nucleolar RNA SNORA17 [Source:RFAM;Acc:RF00560] |
| 44 | ENSMUSG00000038736 | Nudcd1 | KNOWN | NudC domain containing 1 [Source:MGI Symbol;Acc:MGI:1914679] |
| 45 | ENSMUSG00000022338 | Eny2 | KNOWN | enhancer of yellow 2 homolog (Drosophila) [Source:MGI Symbol;Acc:MGI:1919286] |
| 46 | ENSMUSG00000094551 | U6 | NOVEL | U6 spliceosomal RNA [Source:RFAM;Acc:RF00026] |
| 47 | ENSMUSG00000038725 | Pkhd1l1 | KNOWN | polycystic kidney and hepatic disease 1-like 1 [Source:MGI Symbol;Acc:MGI:2183153] |
| 48 | ENSMUSG00000022339 | Ebag9 | KNOWN | estrogen receptor-binding fragment-associated gene 9 [Source:MGI Symbol;Acc:MGI:1859920] |
| 49 | ENSMUSG00000022340 | Sybu | KNOWN | syntabulin (syntaxin-interacting) [Source:MGI Symbol;Acc:MGI:2442392] |
| 50 | ENSMUSG00000097102 | AC100730.1 | KNOWN | NA |
| 51 | ENSMUSG00000060530 | A930017M01Rik | KNOWN | RIKEN cDNA A930017M01 gene [Source:MGI Symbol;Acc:MGI:2685151] |
| 52 | ENSMUSG00000089137 | U6 | NOVEL | U6 spliceosomal RNA [Source:RFAM;Acc:RF00026] |
| 53 | ENSMUSG00000089589 | SNORA48 | NOVEL | Small nucleolar RNA SNORA48 [Source:RFAM;Acc:RF00554] |
| 54 | ENSMUSG00000058463 | Gm5471 | KNOWN | predicted pseudogene 5471 [Source:MGI Symbol;Acc:MGI:3648310] |
| 55 | ENSMUSG00000022342 | Kcnv1 | KNOWN | potassium channel, subfamily V, member 1 [Source:MGI Symbol;Acc:MGI:1914748] |
| 56 | ENSMUSG00000096157 | AC125523.1 | NOVEL | NA |
| 57 | ENSMUSG00000065312 | U6 | NOVEL | U6 spliceosomal RNA [Source:RFAM;Acc:RF00026] |
| 58 | ENSMUSG00000092643 | AC124746.1 | NOVEL | NA |
| 59 | ENSMUSG00000051198 | 4930548G14Rik | KNOWN | RIKEN cDNA 4930548G14 gene [Source:MGI Symbol;Acc:MGI:1922531] |
| 60 | ENSMUSG00000088679 | U7 | NOVEL | U7 small nuclear RNA [Source:RFAM;Acc:RF00066] |
| 61 | ENSMUSG00000022311 | Csmd3 | KNOWN | CUB and Sushi multiple domains 3 [Source:MGI Symbol;Acc:MGI:2386403] |
| 62 | ENSMUSG00000089828 | Gm16300 | KNOWN | predicted gene 16300 [Source:MGI Symbol;Acc:MGI:3826587] |
| 63 | ENSMUSG00000077752 | SNORA17 | NOVEL | Small nucleolar RNA SNORA17 [Source:RFAM;Acc:RF00560] |
| 64 | ENSMUSG00000065742 | U2 | NOVEL | U2 spliceosomal RNA [Source:RFAM;Acc:RF00004] |
| 65 | ENSMUSG00000089314 | U6 | NOVEL | U6 spliceosomal RNA [Source:RFAM;Acc:RF00026] |
| 66 | ENSMUSG00000088013 | U7 | NOVEL | U7 small nuclear RNA [Source:RFAM;Acc:RF00066] |
| 67 | ENSMUSG00000088023 | U7 | NOVEL | U7 small nuclear RNA [Source:RFAM;Acc:RF00066] |
| 68 | ENSMUSG00000065760 | U6 | NOVEL | U6 spliceosomal RNA [Source:RFAM;Acc:RF00026] |
| 69 | ENSMUSG00000038679 | Trps1 | KNOWN | trichorhinophalangeal syndrome I (human) [Source:MGI Symbol;Acc:MGI:1927616] |
| 70 | ENSMUSG00000084458 | Mir1907 | KNOWN | microRNA 1907 [Source:MGI Symbol;Acc:MGI:3811422] |
| 71 | ENSMUSG00000022312 | Eif3h | KNOWN | eukaryotic translation initiation factor 3, subunit H [Source:MGI Symbol;Acc:MGI:1915385] |
| 72 | ENSMUSG00000022313 | Utp23 | KNOWN | UTP23, small subunit (SSU) processome component, homolog (yeast) [Source:MGI Symbol;Acc:MGI:1925831] |
| 73 | ENSMUSG00000022314 | Rad21 | KNOWN | RAD21 homolog (S. pombe) [Source:MGI Symbol;Acc:MGI:108016] |
